# Role of the staphylococcal nuclease and tudor domain containing 1 in oncogenesis (Review)

**DOI:** 10.3892/ijo.2014.2766

**Published:** 2014-11-18

**Authors:** NIDHI JARIWALA, DEVARAJA RAJASEKARAN, JYOTI SRIVASTAVA, RACHEL GREDLER, MAAGED A. AKIEL, CHADIA L. ROBERTSON, LUNI EMDAD, PAUL B. FISHER, DEVANAND SARKAR

**Affiliations:** 1 Department of Human and Molecular Genetics, Virginia Commonwealth University, Richmond, VA, USA; 2 Department of Biochemistry, Virginia Commonwealth University, Richmond, VA, USA; 3 VCU Institute of Molecular Medicine, Virginia Commonwealth University, Richmond, VA, USA; 4 VCU Massey Cancer Center, Virginia Commonwealth University, Richmond, VA, USA

**Keywords:** staphylococcal nuclease and tudor domain containing 1, astrocyte elevated gene-1, cancer, metastasis

## Abstract

The staphylococcal nuclease and tudor domain containing 1 (SND1) is a multifunctional protein overexpressed in breast, prostate, colorectal and hepatocellular carcinomas and malignant glioma. Molecular studies have revealed the multifaceted activities of SND1 involved in regulating gene expression at transcriptional as well as post-transcriptional levels. Early studies identified SND1 as a transcriptional co-activator. SND1 is also a component of RNA-induced silencing complex (RISC) thus mediating RNAi function, a regulator of mRNA splicing, editing and stability, and plays a role in maintenance of cell viability. Such diverse actions allow the SND1 to modulate a complex array of molecular networks, thereby promoting carcinogenesis. Here, we describe the crucial role of SND1 in cancer development and progression, and highlight SND1 as a potential target for therapeutic intervention.

## 1. Introduction

Cancer is a highly aggressive disease characterized by interplay of mutations that cause cellular transformation and abnormal cell growth. Over the years, state-of-the-art studies have provided significant insights into the pathogenesis of this highly complex disease. Cancer is now well-defined by six hallmarks: sustained proliferative signaling, evasion of growth suppression, invasion and metastasis, replicative immortality, angiogenesis and resistance to apoptosis (1). Changes in expression patterns of any gene controlling these pathways initiate tumorigenesis that has the potential of developing malignancy. With increasing number of mutations being identified each day, that play a role in cancer development, molecular understanding of this disease gets more complicated. Further adding to the complexity is the effect of environmental factors in disease pathogenesis at each stage. Mutations arising in cells are mainly governed by genetic predisposition to changes in DNA structure, stability and expression, epigenetic changes and environmental factors (2). Epigenetic variations broadly include methylation pattern, microRNA expression disorders and chromatin organization (2). Diversity in cancer is not just restricted to the factors leading to cancer development, but is also affected by the tumor microenvironment, broadly classified as intertumor heterogeneity and intra-tumor heterogeneity (2). With the advent of new technologies, the molecular changes, which are responsible for such alterations, are being identified and a subset of these can serve as potential therapeutic targets.

The staphylococcal nuclease and tudor domain containing 1 (SND1) is involved in transcriptional activation, RNA splicing, editing and stability, and RNAi function (3–10). These processes are relevant for regulation of gene expression. SND1 is predicted to manifest a dynamic role modulating multiple molecular networks that control gene expression. Structural analysis has confirmed that the SND1 protein functions include nucleic acid interaction along with protein-protein interactions (11). Several studies have illustrated the significant association of SND1 with different types of cancers, including liver, breast, prostate and colorectal cancers and malignant glioma (12–20). It is overexpressed in these cancers and known to promote manifestation of aforementioned hallmarks of carcinogenesis. The present review provides a comprehensive description of the functional aspects of SND1 that are relevant to cancer development and progression.

## 2. SND1 protein structure

The human SND1 gene was assigned to chromosome 7q31.3 band location employing restriction fragment and fluorescence *in situ* hybridization (FISH) analyses (21). Genomic gain in 7q, especially 7q31, has been demonstrated in prostate, renal and colorectal carcinoma (22–24). SND1 is overexpressed in prostate and colorectal carcinoma (18,19) and genomic amplification might be an underlying mechanism for this overexpression.

**Figure 1:**
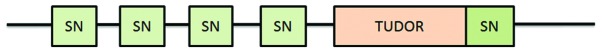
The domain structure of SND1.

**Figure 2:**
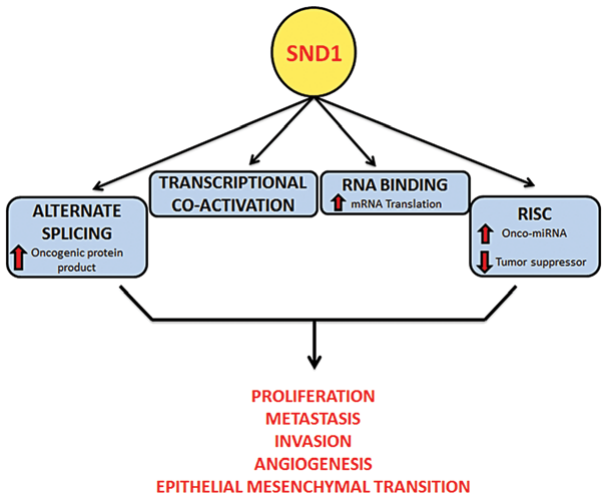
Schematic representation of SND1 functions contributing to oncogenesis.

Human SND1 is a 910-a.a. containing protein with highly conserved domains found even in *Caenorhabditis elegans*. SND1 comprises tandem repeats of four nuclease (SN) domains and a fifth domain containing fusion of Tudor and partial nuclease domains (TSN) (11) (Fig. 1). The nuclease domains share 20–30% sequence homology (3). These domains have been well characterized and found to be structurally related to staphylococcal nuclease (SN) domains (3). These are thermonucleases that hydrolyze DNA and RNA in a calcium-dependent manner (3). However, hydrophobic cluster analysis shows that the SN domains of SND1 lack the specific amino acid residues involved in calcium-dependent catalytic activity (3). SN domains are included in the oligonucleotide/oligosaccharide binding (OB) fold superfamily, which comprises a large number of proteins, involved in nucleic acid binding (3). OB fold proteins are critical for DNA replication, DNA recombination, DNA repair, telomeric maintenance and cold shock response (25). OB fold domains range from 70 to 150 a.a. in length and consist of variable loops between conserved secondary domains (25). Other than staphylococcal nucleases, OB fold is also observed in bacterial enterotoxins, nucleic acid binding proteins and inorganic pyrophosphates (25). Many of the proteins in OB fold family, lack catalytic activity but carry out several other functions, such as transcriptional activation or repression, chromatin modification and DNA repair (3,25). Tudor domains are highly conserved domains across eukaryotic species and studied in great detail employing *Drosophila* as a model system (26). Tudor domain containing proteins are involved in DNA interactions, specifically in epigenetic regulation, gene expression as well as snRNP, miRNA and siRNA biogenesis (26). The presence of these versatile SN and Tudor domains confer SND1 with its diverse multifunctional properties (Fig. 2).

## 3. Regulation of SND1 expression

The isolated 5′-flanking region of human SND1 gene spans >3.8 kb, including transcription start site and translation initiation codon (27). Bioinformatics analysis revealed presence of putative binding motifs for NF-κB, Sp1 and NF-Y transcription factors (27). The core promoter region does not contain TATA box, but is rich in CpG islands to form pre-initiation transcription complexes. The lack of a TATA box, an initiator sequence for transcription, is complimented by the presence of a CCAAT box in the reverse orientation, i.e., ATTGG at position −61 and −28 (27). The CCAAT box and a GC box, at −48, act as positive regulatory elements for SND1 expression (27). Inverted CCAAT sequences are often binding targets for the NF-Y transcription factor. Luciferase assays with promoter deletion mutants show that −274 to −112, containing potential binding sites for NF-κB and Sp1, are crucial for SND1 expression (27). Interaction of NF-κB, Sp1 and NF-Y with the SND1 promoter was also detected by chromatin immnunoprecipitation (27). Mutation in GC box or CCAAT box reduced SND1 expression by 55–75%. As expected, TNFα, an activator of NF-κB, induced SND1 transcription, thereby suggesting the functional role of NF-κB in SND1 transcription regulation (27).

Promoter regions of the human, mouse and rat SND1 gene show approximately 80–85% sequence homology (27). Similar findings have been observed in promoter analysis of rat homologue of SND1, p102 (28,29). Putative binding sites for CCAAT/enhancer binding protein (C/EBP), STATs and upstream stimulatory factor (USF) have been identified (28). NF-Y binds at the CCAAT box (−370, −366), whereas the GC rich regions were identified as putative Sp1 binding sites (29). DNA elements that matched the consensus binding sequences for liver specific transcription factors, such as HNF-4, were also identified (28), suggesting that SND1 might be differentially expressed in a tissue-specific manner.

Fashe *et al* investigated cellular localization and tissue specific expression of SND1 in mice. With an exception of muscle tissues, SND1 is ubiquitously expressed in mice and the expression patterns are mostly consistent to that reported in humans (30). SND1 is noticeably upregulated in active secretory organs including pancreas, liver and mammary glands (30). Studies have reported upregulated levels of SND1 in lipid droplets of milk secreting mammary epithelial cells (31). SND1 levels were higher in exocrine pancreatic cells as compared to endocrine cells (30). SND1 expression is lower in hepatocytes, however, it is overexpressed in sinusoid endothelial cells (30). Actively proliferating cells such as crypts of Lieberkuhn and basal keratinocytes of skin and hair follicles show high SND1 protein levels compared to more differentiated or terminally differentiated cells in the same tissue. SND1 protein levels are much higher in spermatogonial cells than in spermatocytes and Sertoli cells. No protein is detected in terminally differentiated spermatids and mature spermatozoa. In ovary, SND1 levels are high in follicular cells compared to that in stromal cells. There is no SND1 protein in oocytes. SND1 protein levels are moderately expressed in human brain tissue, in neuronal as well as glial cells. In mouse kidney, SND1 is comparatively overexpressed in endothelial cells of Bowman’s capsule than podocytes and mesangial cells. This protein is also expressed in bronchiolar epithelium, alveolar cells and pneumocytes of lung. Within gastrointestinal system, SND1 is expressed in ileum, deuodenum and colon. Lower SND1 levels are observed in more differentiated cells like villi and Paneth cells in the ileum and absorptive cells of the colon. Overall, SND1 is overexpressed in rapidly proliferating or precursor cells and downregulated in terminally differentiated cells (30). SND1 is upregulated in T-cells and co-localizes with CD3, as observed in lymphoid organs (30). SND1 expression is not evident in macrophages, in lymphoid organs or in tissue residing macrophages like Kuppfer’s cells in liver and alveolar macrophages in lungs. Further, SND1 levels in the red pulp of spleen are found to be higher than that in white pulp. Differential expression of SND1 in T lymphocytes and macropahges suggests a potential role of SND1 in regulating immunity.

## 4. Functions of SND1

### Regulation of transcription

SND1, also called TudorSN or p100, was first identified as a transcriptional co-activator in an attempt to identify proteins interacting with Epstein-Barr nuclear antigen (32). EBNA2 specifically activates transcription of genes that mediate B lymphocyte transformation (32). Along with known interacting factors such as TFIIB, TFIIH and TAF40, SND1 specifically interacts with EBNA2 acidic domain (32). This interaction is mediated by SND1-TFIIE interaction such that SND1 acts as an adapter protein between EBNA2 and the transcriptional machinery (32). Pim kinase, found to be upregulated during Epstein-Barr virus infection, also interacts with SND1 and mediate cellular transformation by cooperating to enhance c-Myb activity (9). It was illustrated that Pim-1 phosphorylates SND1 and forms a stable complex, leading to an induction in c-Myb activity (9). Ectopic expression of Ras and Pim-1 also induce c-Myb responsive genes, since c-Myb is a downstream target in this signaling cascade (9). This research study thus revealed that SND1 is a vital link in Ras- and Pim-1-mediated induction of c-Myb (9). Lack of SND1 or dominant negative alleles of SND1 failed to cause an induction in c-Myb activity (9). c-Myb is linked to proliferation and differentiation, and also known to mediate cellular transformation. Hence, a role of SND1 in regulating c-Myb expression via Ras or Pim-1 is significant in understanding its functional impact in oncogenesis.

Another important class of transcription factors that are known to interact and cooperate with SND1 is the signal transducers and activator of transcription, i.e., STATs (4,8,33). STAT proteins are sequestered to Janus kinases (JAK), which upon stimulation by cytokines like IFN-γ, undergo phosphorylation (34). Phosphorylation of STAT proteins and subsequent dimerization leads to nuclear localization where it activates transcription of target genes (34). Constitutive activation of the JAK-STAT pathway has been implicated in many cancers (34). There are seven STAT proteins, which share structural and functional homology. Of these, SND1 is known to act as a transcriptional co-activator for STAT5 and STAT6. SND1 co-immunoprecipitates with STAT5, confirming the protein-protein interaction, but does not affect the phosphorylation status of STAT5 (8). Interaction with STAT5 is mediated via the SN domains as well as Tudor domains of SND1, whereas STAT6-SND1 interactions involve only SN domains. SND1 acts as an adapter molecule, allowing functional bridging between CREB binding proteins (CBP) and STAT6 (33). It interacts with the D3-D4 domains of CBP and STAT6 transactivation domain (TAD). Histone acetylase activity of CBP is stimulated by SND1, thereby allowing transcriptional activation (33).

A recent study in a breast cancer model demonstrated that SND1 significantly interacts with the promoter regions of several genes in the TGFβ signaling pathway, including Smad 1–4 and TGFβ (35). We have shown that SND1 upregulation also correlates with TGFβ signaling in HCC, as described later in this review (16).

### Post-transcriptional regulation of gene expression

#### Regulation of RNA-induced silencing complex (RISC) activity

Post-transcriptional regulation of gene expression can be mediated by several mechanisms including nucleocytoplasmic localization, mRNA stability, mRNA processing and translation. SND1 functions as a nuclease in RNA-induced silencing complex (RISC) that plays a significant role in modulation of gene expression at a post-transcriptional level (6). Our studies have shown that astrocyte elevated gene-1 (AEG1), an important oncogene (36,37), in association with SND1 and other proteins, forms a stable RISC complex (12). RISC incorporates one strand of a small interfering RNA (siRNA) or microRNA (miRNA) and uses the siRNA or miRNA as a template for recognizing complementary mRNA. Argonaute proteins are activated in RISC when a complementary mRNA is identified which then cleaves the mRNA. SND1 functions as a nuclease in RISC along with the Argonaute proteins, while AEG-1 functions as a scaffold protein for proper assembly of this complex. Both SND1 and AEG-1 are overexpressed in multiple cancers and together they facilitate functions of oncogenic miRNAs (onco-miRNA). Indeed RISC activity in cancer cells was found to be higher than that in normal cells (12). The increased activity of onco-miRNAs leads to increased suppression of their target tumor suppressor genes. In HCC cells it was documented that overexpression of AEG-1 or SND1 resulted in decreased expression of several tumor suppressor genes that are targets of oncomiRNAs, e.g., PTEN, target of miR-221 and miR-21; CDKN1C (p57), target of miR-221; CDKN1A (p21), target of miR-106b; SPRY2, target of miR-21; and TGFBR2, target of miR-93 (12). The reverse finding was observed upon knockdown of SND1 or AEG-1 (12). These findings suggest that increased RISC activity conferred by SND1 and AEG-1 might contribute to the carcinogenic process (12).

In pancreatic cancer, synaptogamin-11 interacts with RISC via SND1 binding (38). It is hypothesized that this protein is the missing link between membrane trafficking and miRNA-mediated gene regulation (38). Synthesis of mature mir17–92 cluster is also inhibited by SND1 protein, thereby affecting several downstream target genes (39).

### Regulation of mRNA stability

SND1 interaction with mRNA transcript can be independent of the RISC. Studies show that SND1 interacts with 3′UTR of angiotensin II type 1 receptor (AT1R), a G protein coupled receptor mediating the action of angiotensin (7). Here, SND1 increases mRNA stability and translational efficiency by increasing AT1R mRNA half-life, resulting in elevated protein levels (7). A recent finding shows that SND1 and AT1R mRNA colocalize in stress granules followed by oxidative stress and SND1 is required for efficient protein-RNA aggregation (40). These findings imply that SND1 increases stability of specific mRNAs, crucial for cellular stress response (40). Using HCC cell lines, we have demonstrated that SND1-mediated increased activity of AT1R activates the TGFβ signaling cascade thereby promoting epithelial-mesenchymal transition (EMT) and an increase in migration and invasion (16). Studies have also established interaction of SND1 and Dengue virus 3′UTR, leading to increased viral replication (41). Further studies are required to determine whether SND1 plays a similar role in promoting replication of hepatitis B or C viruses (HBV or HCV), the most common cause of HCC.

### Regulation of mRNA splicing

Splicing is an important post-transcriptional event, involved in excluding the non-coding intronic regions of mRNA transcript and thereby allowing translation of exonic regions into a functional polypeptide (42). A large macromolecular complex, driven by several proteins is required for this processing. Splicing is tightly regulated and functionally coupled with transcription (42). Because differential regulation of genes influencing cell growth and proliferation are critical for carcinogenesis, splicing is speculated to play a vital role in establishing pathogenesis (43). The spliceosome complex is comprised of five major small ribonucleoproteins - U1, U2, U4/U6 and U5 along with several small non-snRNP (42,44). SND1 interacts with the U5 component of the spliceosome and other non-snRNPs (44). Immunoprecipitation studies with GST-TSN fusion protein (lacking SN domains) and GST-SN fusion protein (lacking TSN domain) demonstrated that this interaction occurs specifically via TSN domain (44). *In vitro*, exogenously added SND1 accelerated the kinetics of spliceosome assembly, detected by a ligated mRNA product, in a dose-dependent manner (44). However, no difference was observed in the amount of splicing products with or without SND1 (44). SND1 improved the efficiency of pre-spliceosomal complex assembly and accelerated the formation of complex B and complex C (44).

Alternative splicing, observed in eukaryotes, allows translation of multiple polypeptides from the same gene transcript by selection of specific exons to be included in the processed mRNA (43). Recent reports indicate that deregulation of alternative splicing is associated with cancer development and progression (43). SND1 plays a role in this biological process. SND1 is identified as an interacting partner for SAM68, a prooncogenic RNA binding protein that is upregulated in prostate cancer and supports cellular proliferation (45). SAM68 is involved in alternative splicing of CD44, specifically favoring inclusion of exon v5 of this gene (45). Prior studies indicate that inclusion of variable exons (v5) in CD44 mRNA correlates with cancer development (45). SND1 functions as a positive regulator of the alternative splicing of CD44 via SAM68. Knocking down SND1 inhibits inclusion of upstream variable exons (v4, v5 and v7), whereas downstream variable exons (v8-v10) and constitutive exons are not affected (45). These findings suggest that SND1 co-ordinates transcriptional and post-transcriptional events to regulate gene expression. Future studies focused on splicing activity of SND1 might uncover insights into the molecular events governing malignancy.

### Regulation of RNA editing

Adenosine deaminase (ADAR) proteins function in RNA A-to-I editing by deaminating Adenosine to Inosine, which is read as guanosine on an mRNA transcript (46). Such processes regulate protein translation by functionally changing the mRNA sequence (46). This post-transcriptional regulation also plays a role in gene expression and miRNA processing (46). Interestingly, ADAR protein expression is tightly regulated during embryogenesis, where it is highest in the oocytes and zygote and diminished in the embryo stages (10). A strong correlation was observed between ADAR and SND1 levels during mouse fertilization (10). While ADAR marks hyper-edited transcripts, SND1 is responsible for degradation of hyper-edited mRNA transcripts as well as miRNA precursors (10). The data suggests that SND1 and ADAR1 are functionally synchronized in A-to-I editing and eliminate most miRNA precursors, progressively from oocyte to zygote (10). However, this function is prohibited during embryogenesis. Thus, SND1 plays a crucial role in early stage embryogenesis and cell differentiation (10). Analysis of a SND1 knockout mouse will provide insights into the role of SND1 in regulating gene expression during the developmental stage.

### SND1 and stress response

SND1 plays a key role in cellular stress response via stress granule formation (47). Several environmental stimuli can be stressful for cellular growth. In response to such stimuli, cells undergo reprogramming in gene expression that allows cell survival (47,48). Initial studies illustrated that SND1 is a component of the cytoplasmic stress granules and the SN domain is crucial for this function (47,48). SND1 interacts with Ras GTPase activating protein SH3 domain binding protein (G3BP) and adenosine deaminase (ADAR1). While G3BP has been shown to be essential for assembly of stress granules (47), ADAR1 has been linked to apoptosis and stress response (48). ADAR1 levels are also induced by interferon and its deficiency causes defective hematopoiesis (48). Such studies implicate ADAR1 in cell survival and immune response towards stress. SND1 was found to directly interact with ADAR1. These proteins co-localized within cytoplasmic stress granules following oxidative stress, heat shock or interferon induction (48). Knockdown of SND1 prohibited assembly of smaller stress foci into larger granules, emphasizing its role in this cellular function (47). These studies need to be extended *in vivo* using appropriate experimental models to confirm the essential role of SND1 to cope with stress responses.

### Regulation of cell viability

SND1 is an essential protein in normal programmed cell death, a process mediated by caspases (49). SND1 is cleaved by caspase 3 during drug-induced apoptosis. A non-cleavable SND1 mutant increased cell viability and knocking down SND1 promoted drug-induced apoptosis in HeLa cells (49). Incubation with caspases completely blocked RNase activity of SND1 indicating that SND1 enzymatic activity is required for maintaining cell viability or protection from apoptosis (49).

## 5. Oncogenic functions of SND1

### Role of SND1 in hepatocellular carcinoma (HCC)

HCC is the primary liver malignancy, characterized as a highly aggressive form of cancer (50). In most cases, HCC develops over a preexisting condition, such as liver cirrhosis, non-alcoholic steatohepatitis and viral hepatitis infection (50). It is the fifth most common cancer and ranks third in cancer-related deaths worldwide (50). Current management options include surgical resection, and transarterial chemoembolization (TACE), though there is virtually no cure for this cancer (51). Molecular approaches such as the multikinase inhibitor Sorafenib help to moderately increase the survival of HCC patients (52). To curb the increasing mortality rate, there is an urgent need for identification of potential therapeutic targets that can be employed for translational applications.

Studies demonstrate multiple ways by which SND1 contributes to hepatocarcinogenesis. Immunohistochemical analysis has demonstrated that SND1 is overexpressed in a high percentage of HCC patients and SND1 levels correlate with HCC stage (12). Stable clones of human HCC cells, with either overexpression or knockdown of SND1, in nude mouse xenograft studies confirmed the positive role of SND1 in regulating cell viability and growth (12). Since SND1 is also an integral component of RISC complex, we investigated if RISC activity correlated with HCC development. Increased RISC activity was observed in HCC cell lines, which consequently led to an increased downregulation of tumor suppressors via RNA interference (12). As pointed out earlier, SND1 interacts and cooperates with AEG-1 to form RISC along with other known RISC proteins and inhibition of SND1 activity diminished the AEG-1 activity (12). Further investigation established that increased SND1 protein levels trigger a cascade of molecular events that promote invasion, proliferation, migration and angiogenesis (12,16,17). We unraveled a linear pathway in which NF-κB activation by SND1 augments miR-221 levels (17). Target genes of miR-221, Angiogenin and CXCL16, were observed to promote angiogenesis (17). Analysis of global gene expression profiles of SND1 knockdown HCC clones identified 123 genes drastically downregulated, many of which are in the TGFβ signaling pathway (16). We documented that increased stabilization of AT1R mRNA by SND1 activates AT1R downstream signaling, such as activation of ERK1/2, culminating in activation of the TGFβ signaling pathway. The TGFβ signaling pathway closely regulates EMT (16). Indeed, *in vitro* analysis demonstrated increased invasion and migration in SND1-overexpressing clones and vice versa in SND1 knockdown clones (16) and expression of EMT marker proteins, such as N-cadherin, Slug and Snail and Vimentin, was found to be congruent with our hypothesis (16). A tissue microarray on 50 HCC cases showed statistically significant correlation between AT1R and SND1 levels further establishing a causative relationship between SND1, AT1R and TGFβ in hepatocarcinogenesis (16).

IGF pathways are reported to be deregulated in HCC and found to be a cause of the aggressive tumorigenesis. Insulin-like growth factor binding protein 3, a negative regulator of IGF pathway, was reported to be significantly overexpressed in SND1 knockdown HCC clones (53). Thus SND1 might contribute to HCC by inhibiting IGFBP3 and promoting IGF-1 activity (53).

### SND1 promotes invasion and metastasis in breast cancer

Cancer cells possess the ability to invade circulatory system and establish tumors in tissues distant from the primary site of tumorigenesis (54). Angiogenesis enables them to acquire nourishment and growth factors for constitutive proliferation. Metastatic potential of a tumor is often a measure of the fatality of the cancer. Metastasis has been long known to be a major cause of disease relapse and cancer related deaths. More patients succumb to metastatic malignancy than by the primary cancer. An efficient prognosis and diagnostic tool has not been discovered, in spite of several attempts at analyzing differential expression patterns in tumorous tissue.

iTRAQ based proteomic analysis of a breast cancer metastasis model revealed that SND1 levels are upregulated with carcinoma progression (13). This investigation aimed at studying gene signatures in the breast cancer metastasis model. Out of the 197 proteins differentially regulated in cancerous tissue as compared to normal tissue, only those that have not been reported in association with metastasis were prioritized for further scrutiny (13). This approach helped identify 10 novel proteins significantly associated with metastasis, employing Mass spectrometry and immunohistochemistry. SND1 was one of these proteins, deregulated in breast cancer metastasis and showed significant differential expression in normal versus breast cancer tissue (13). Immunohistochemical analysis of a tissue microarray showed upregulation of SND1 in the majority of the cases (13).

AEG-1, also called Metadherin, is a known oncogene associated with most oncogenic phenotype such as metastasis, invasion, angiogenesis, chemoresistance and apoptosis (55–59). A study aimed at exploring role of AEG1 in breast cancer metastasis, identified SND1 as an AEG-1 interacting protein (14). Role of SND1 in metastasis was further emphasized based on the global gene expression profiling of SND1 knockdown clones. The list of gene sets globally enriched in SND1-expressing control cells versus SND1-KD cells was strikingly dominated by those involving genes upregulated in some component of metastatic or oncogenic signaling (14). Genes such as ANGPTL, ID1 and EREG, known to promote specifically lung metastasis in breast cancer model, were significantly enriched in this gene set (14). An experimental lung metastasis study in nude mice using a highly metastatic breast cancer cell line with SND1 knock down showed dramatic reduction in pulmonary metastatic burden *in vivo* (14). Although SND1 did not promote invasion, it was found to augment chemoresistance to cells (14). SND1-KD cells demonstrated sensitivity towards chemotherapeutic drug-induced apoptosis. Microarray data from SND1-KD cells indicated upregulation of the KiSS1 gene (14). Reporter assays later confirmed that SND1 directly downregulates the expression of the KiSS1 gene that is known to suppress metastasis (60). Analysis of clinical data set of breast cancer patients with metastasis revealed that SND1 levels strongly correlate with lung metastasis incidence and metastasis-free survival (14). Thus, SND1 is established to be a pro-metastasis protein. A recent finding shows significant relationship between AEG-1-SND1 interactions and mammary tumorigenesis (15). AEG-1 knockout cells show reduced tumor initiation and sphere formation *in vivo* (15). This effect could be completely rescued by ectopic expression of AEG-1 in these cells (15). However, knocking down SND1 in these cells completely abolished the rescue effect of ectopic AEG-1 (15). Knocking down SND1 in AEG1^+/+^ cells reduced the sphere formation *in vitro* and tumor formation *in vivo*, resembling the phenotype of AEG1^−/−^ cells (15). Thus, SND1 is essential for pro-oncogenic properties of AEG-1 in breast cancer (15).

Early studies have established that SND1 cooperates with c-Myb, a differentiation and growth factor for immature hematopoietic stem cells promoting lymphocyte transformation (9). Studies in breast cancer patients show overexpression of c-Myb as well as SND1 (61). A study focused on identifying target genes of c-Myb revealed that the SND1 promoter is one of the target sites of c-Myb (61). There might be a positive regulatory mechanism to maintain levels of c-Myb and SND1 that potentially maintain tumorigenesis in breast cancer.

### SND1: an efficient diagnostic marker for prostate cancer and colorectal cancer

Studies focused on prostate cancer have identified SND1 as an efficient diagnostic marker (18). In 174 prostate cancer patients, SND1 levels correlated with histological grade of the tumor (18). SND1 protein levels were comparable to α-methylacyl-coA racemase (AMACR) protein levels, a currently employed marker protein for prostate cancer diagnosis (18). Though there were some cases where SND1 and AMACR protein levels differed, there was robust overexpression of SND1 in tumorigenic tissue (18,45). It was suggested that multiple protein markers, including SND1 and AMACR, should be used for better diagnosis of the disease (18). Knocking down SND1 reduced proliferation of prostate cancer cells demonstrating the importance of SND1 in maintaining prostate cancer viability (18). SND1 also promotes prostate cancer development by positively regulating CD44 alternative splicing, allowing inclusion of variable exon v5 that is known to be pro-oncogenic (45).

SND1 is significantly associated with colorectal cancer. A recent genome wide analysis of methylation patterns in CRC patients revealed a pool of genes that are differentially methylated in cancerous tissue in comparison to adjacent normal tissue (62). The study also included tissue from normal, healthy patients with no familial history of CRC, as a control. A CpG site located in SND1 gene was identified with highest discriminative accuracy, highlighting role of this protein in oncogenesis (62). Most of the hypermethylated CpG sites were within the SND1 gene regions, whereas methylation in the promoter region of SND1 was observed to be comparatively lower. Accordingly, SND1, along with other genes was identified as a potential diagnostic candidate gene (62). Significant correlation was observed in nodal stage, pathological stage and co-expression of SND1 and AEG-1 in colon cancer (63). Immunohistochemical analyses of 196 colon cancer cases establish that cytoplasmic expression of AEG1 and SND1 protein positively correlates with tumor grade and cancer progression, but negatively correlates with post-operative survival of patients (63). The study suggests potential application of these proteins as prognostic markers for colon cancer (63).

SND1 downregulates adenomateous polyposis coli protein levels by post-transcriptional modification, without altering the mRNA levels of this gene, as reported in colon cancer (19). APC is a tumor suppressor in colon carcinogenesis and also associated with maintaining cell polarity and cell-cell adhesion by regulating E-cadherin localization (19). Loss of APC protein leads to stabilization and subsequent accumulation of β-catenin by preventing its proteasomal degradation with subsequent loss of contact inhibition and increased proliferation (19). These findings suggested that SND1 might be involved in early stage colon carcinogenesis. However, whether miRNAs or RISC is involved in SND1-mediated downregulation of APC protein remains to be studied.

### SND1 in malignant glioma

Malignant gliomas are the most frequent malignant brain tumor in adults (64). Current multi-modality therapies include surgery, radiation and chemotherapy, nonetheless the prognosis of malignant glioma remains extremely poor (65). The rapid growth and highly invasive nature of malignant glioma, favors its infiltration into surrounding normal brain parenchyma and facilitates recurrence after therapy (66). This aggressive disease progression necessitates further understanding of molecular mechanism involved in glioma growth and invasion that ultimately will lead to identification of novel therapeutic targets of translational relevance.

Our recent studies suggest that SND1 may provide a novel target for malignant glioma treatment (20). In this study we found higher SND1 mRNA and protein in human malignant glioma tissue as compared to normal brain. TCGA data showed a similar trend of high SND1 expression in human astrocytoma and glioblastoma samples as compared to normal brain. Additionally Rembrandt data (https://caintegrator.nci.nih.gov/Rembrandt) supports the prognostic significance of SND1 expression in which patients with intermediate levels of SND1 survived longer than patients showing elevated SND1 expression. Overexpression (OE) and knock down (KD) studies were performed to unravel the functional significance of SND1 in glioma progression. Overexpression of SND1 in immortalized primary human fetal astrocytes (IM-PHFA) (low SND1) significantly enhanced invasion and colony formation as compared to parental IM-PHFA cells. Conversely when SND1 was knocked down in multiple glioma cell lines (high SND1) it significantly decreased invasion and colony formation, both in monolayer and in soft agar. Interestingly SND1-KD primary glioma cells demonstrated enhanced sensitivity towards Temozolomide, an FDA approved drug used with radiation therapy as a standard of treatment for GBM patients (67). Knock down of SND1 in malignant glioma cells resulted in a flat-shaped cells, which stained positive with β-galactosidase indicating induction of cellular senescence. Further studies documented a potential involvement of STAT-3 in SND-1-mediating glioma invasion and senescence-induced cell death. An intracranial xenograft study in nude mice using a highly invasive patient-derived malignant glioma cell line, showed a significant improved survival in SND1-KD group (20). This was associated with a significant decrease in proliferation marker, activated STAT3, and angiogenesis marker and an enhanced expression of apoptotic marker. The observation that SND1 regulates several important determinants of glioma progression supports the rationale of using SND1-inhibition as a means of treating glioma patients.

## 6. Specific inhibitors of SND1

BLAST search reveals that SND1 is the only eukaryotic protein with a Tudor and SN fusion domain. Hence, this quaternary fold can be employed for targeted therapeutic approaches, developing SND1 specific inhibitors. A similar protein domain is observed in *Plasmodium falciparum*, a parasite causing malarial infections in humans. Studies with *P. falciparum* SND1 have confirmed that the SN domain is involved in nuclease activity, whereas the Tudor domain carries out the function of RNA binding (68). 3′, 5′-Deoxythymidine bisphosphate (pdTp) is a competitive chemical inhibitor against SNases (6). In *P. falciparum*, it not only blocks the nuclease activity, but also inhibits RNA/protein interaction of SND1 (68). pdTp inhibited growth of both chloroquine-sensitive and chloroquine-resistant strains of *P. falciparum* at a concentration of 100–200 μM. Nevertheless, it did not inhibit the growth of human HCC cell line Huh7-D12 at a concentration as high as 800 μM suggesting that pdTp might be developed as an anti-malarial drug (68). However, studies performed in our laboratory using multiple human HCC cell lines demonstrated that pdTp treatment resulted in significant reduction in cell viability and colony forming potential (12). This effect was observed at high concentrations, ranging from 100 to 200 μM thus rendering it ineffective for clinical applications. Further high throughput screening assays need to be performed to develop clinically relevant chemical inhibitors against SND1 enzymatic activity. Employing the C terminus fusion domain of SND1 might prove to be a productive effort in designing targeted molecular therapy.

## 7. Conclusion and future direction

SND1 is known to orchestrate a series of changes that affect global gene expression and confer cellular transformation. It regulates gene expression employing multiple unique mechanisms that function at transcriptional as well as post-transcriptional levels. With increasing relevance of studies on SND1 in the context of carcinogenesis it is important to elucidate the molecular mechanisms underlying SND1 activity. Studies so far suggest that it is a promising molecule for clinical investigation and targeted therapeutic management of cancer. Considering the pleiotrophic functions of SND1, one important question that has not been addressed is the role of SND1 in maintaining normal physiological function(s) including growth and development. An SND1 knockout mouse, conditional and global, will not only provide comprehensive insights into the physiological functions of SND1, but also will be an ideal model to interrogate the role of SND1 in immortalization, transformation, metastasis and overall cancer development and progression. Conversely, organ-specific SND1 transgenic mouse will also provide useful insight into the oncogenic function of overexpressed SND1. Is SND1 overexpression alone sufficient for transforming normal cells into cancer cells? Can SND1 function as a driver for tumorigenesis or is it a passenger and expediter of transformation following initial mutagenic events? Which particular aspects of SND1 function are most relevant to confer its oncogenic properties? Future studies need to focus on and address these questions, which will provide important insights into the precise role of SND1 in normal and abnormal physiology.
